# Multi-slice CT coronary angiography versus invasive coronary angiography in the assessment of graft patency after coronary artery bypasses graft surgery

**DOI:** 10.1186/s43044-023-00424-8

**Published:** 2023-12-06

**Authors:** Khaled M. Elmaghraby, Salwa R. Demitry, Eman A. Hasaballah, Nady A. Razik

**Affiliations:** Department of Cardiovascular Medicine, Faculty of Medicine, Assiut University, Assiut, 71515 Egypt

**Keywords:** Graft patency, Coronary computed tomography angiography, Coronary artery bypass grafts, Radiation dose in coronary computed tomography angiography

## Abstract

**Background:**

The long-term patency of arterial and venous grafts is crucial for the success of CABG. This study was designed to investigate graft patency using 128-slice coronary computed tomography angiography (CCTA) and compared the results with those obtained using invasive coronary angiography (ICA). In this observational cross-sectional study, we included 40 symptomatic post-CABG patients underwent CCTA and ICA within the same month.

**Results:**

Fifty-five percent were aged more than 60 years, and 80% were males. 67.5% had diabetes, 90% had hypertension, and 30% were smokers. Mean body mass index was 28.89 ± 5.17 kg/m^2^. Mean duration since CABG was 5.25 ± 4.04 years. In total, 124 native vessels and 97 grafts were assessed using CCTA and ICA. CCTA delineated 8 non-cannulated venous grafts and 6 non-cannulated left internal mammary artery grafts. CCTA required a significantly lower radiation dose (1165.77 ± 123.54 vs. 47,589.78 ± 6967.53, *p* < 0.001).

**Conclusion:**

CCTA can be as accurate as ICA in assessing bypass grafts with less radiation dose, providing a non-invasive reliable tool for evaluation.

## Background

Coronary artery bypass grafting (CABG) is the gold standard of treatment of complex and multi-vessel coronary artery disease [1]. Long-term patency of arterial and venous grafts is the most important prognostic factor in post-CABG patients. In current practice, most of CABG procedures are performed using the left internal mammary artery (LIMA) anastomosis to the left anterior descending artery (LAD) and vein grafts used to bypass additional stenosis to perform complete revascularization [2]. Traditionally, graft patency is assessed using invasive coronary angiography (ICA); however, with the introduction of multi-slice computed tomography (MDST), the interest in assessing CABG patients using a non-invasive technique has grown stronger [3].

Symptomatic patients after CABG represent a challenging diagnosis to angiographers. The calcified, diffusely diseased, and tortuous coronary arterial tree limits precise delineation of the lesion. Performing this angiographic evaluation non-invasively is even more challenging. High-quality CT angiographic images and a good understanding of the coronary arterial anatomy are required to properly determine revascularization techniques used [4].

ICA carries the risk of arrhythmia, embolic stroke, dissection of native vessels or grafts, and myocardial infarction. There is up to 2% risk of morbidity (0.43% risk of vascular complications, 0.05% risk of myocardial infarction, 0.07% risk of stroke), and 0.14–0.28% risk of mortality [5]. Furthermore, the technical difficulty encountered due to the variable locations of graft ostia with longer procedure time, more contrast use, radiation exposure, and lower success rates of graft cannulation [6].

However, CCTA is a reliable, non-invasive technique for assessing post-CABG patients as reported by Koplay et al., who revealed that CCTA findings were the same as ICA findings in evaluating stenosis more than 50%, with sensitivity, specificity, and diagnostic accuracy of MSCT were 90%, 99.3%, and 98.7%, respectively [7]. Moreover, Sahiner et al. have studied 284 patients who had 684 bypass grafts, whereas Andreini et al. have investigated 119 patients who had 277 bypass grafts. The previous two studies have reported a high sensitivity, specificity, and negative predictive values greater than 97% [8, 9].

## Methods

### Study design and setting

We conducted our observational prospective cross-sectional study by involving 40 post-CABG patients evaluated using both CCTA and ICA. The study was approved by our Institutional Review Board (no. 17101604) and complied with the Declaration of Helsinki. Confidentiality and privacy of all data were assured. Before conducting the study, informed consent was obtained from each participant after explaining the aim. The research project is registered on ClinicalTrial.gov (registration no. NCT03265041).

### Study participants

All patients who underwent CABG surgery more than 1 year ago, presented to our center, and met the inclusion criteria (eligible for CCTA after a free, informed written consent) were included in this study. The patients with prior CABG were assessed using both CCTA and ICA. Patients with renal insufficiency (serum creatinine level of more than 1.5 mg/dl), congestive heart failure, atrial fibrillation, hypersensitivity to the iodinated contrast agent or history of allergy to certain medications, and those who could not perform breath-hold for 15 s were excluded from the study. We also excluded patients with inadequate CCTA study quality.

### Study variables and data measurements

Included patients were subjected to through history taking which included age; sex; diabetes mellitus (DM); hypertension; smoking; dyslipidemia; heart failure symptoms; renal insufficiency; contraindications to contrast agent or other medications (such as beta-blockers, nitroglycerin); and any prior allergic reactions. Full examination was performed, including general examination; height, weight, body mass index (BMI), blood pressure measurement, heart rate assessment, and cardiac examination to detect any signs of heart failure. The ability to follow breath-hold instructions and perform good inspiratory breath-hold was evaluated. A 12-lead electrocardiogram was performed for all patients. Before CCTA assessment, baseline venous blood samples were collected for evaluating serum creatinine levels and lipid profile. A baseline transthoracic echocardiography study was conducted for all patients (for ejection fraction (EF) estimation as well as cardiac dimensions and valves assessment). All enrolled patients underwent CCTA and ICA within 1 month after the aforementioned tests.

#### CCTA

##### Patient positioning and preparation for CT scanning

Patients were placed in the supine position on the CT examination table. Three ECG leads were connected to obtain an excellent ECG tracing. To synchronize the ECG signal with the raw image data, a noise-free ECG signal was maintained.

A sizable intravenous line (e.g., 18 gauge) was inserted in the right antecubital fossa to allow easy injection of the contrast agent. A 90–120 mL bolus of water-soluble non-ionic contrast (Ultravist 370 mg/mL Schering, Berlin, Germany) was injected through the cannula at a flow rate of 5 mL/s, followed by the injection of approximately 40–50-mL saline at a flow rate of 4 mL/s using a programmed dual-head power injector pump.

The patients were braced for the sensations caused by the injection of the contrast agent as well as repeated breath-hold tests.

##### Image acquisition and reconstruction

All CT scans were done using a 128-slice dual-source CT scanner (Somatom Definition, Siemens, Germany).

The dataset for CCTA was acquired in three steps: topogram, determination of the appropriate time for initiating CCTA image acquisition to secure adequate homogeneous contrast enhancement of all coronary artery tree, and CCTA scan.

Calcium scoring was not performed for all patients. The axial images were obtained using the bolus-tracing technique with a low radiation dose of 120 kV and 40 mAs.

##### ECG gating

Retrospective ECG gating was used depending on the least motion phases for image reconstruction*.*

##### Image evaluation and reconstruction

The scans were analyzed by a CCTA expert (more than 12 years’ of experience in CCTA) using a dedicated workstation that is equipped with specialized cardiac post-processing software (Syngo via, Siemens, Germany). The datasets were evaluated to detect significant coronary stenosis using original axial, multi-planar reformatted images (MPR), maximum intensity projection images (MIP), and volume-rendering techniques.

#### ICA

Blinded to the results of CCTA, one experienced interventional cardiologist analyzed the native coronary segments, all grafts segments with their anastomosis and distal runoffs. Significant stenosis was defined as lesions with more than 50% lumen diameter stenosis in two orthogonal planes.

### Statistical analysis

We performed data entry and data analysis using Statistical Package for the Social Sciences (SPSS version 22; IBM Corp., Armonk, NY, USA). Data were presented as numbers, percentages, means, medians, and standard deviations. The Chi-square and Fisher’s exact tests were used to compare qualitative variables, while to compare quantitative variables, the independent samples t-test for parametric data and the Mann–Whitney test for nonparametric data were used. The kappa test was used to detect agreement between CCTA and ICA. Differences were considered of statistical significance when *p*-values of less than 0.05.

## Results

### Baseline data

We enrolled 40 patients with prior CABG assessed using CCTA and ICA to detect graft patency and native vessel disease progression.

In the study group, most patients were older than 60 years (55%) with a male predominance (80%). Among the population under study, 27 patients had DM (67.5%), 36 patients had hypertension (90%), and 12 patients were smokers (30%). In this study, the mean BMI was 28.89 ± 5.17 kg/m^2^. Regarding the lipid profile results of the study group, the mean LDL level was 84.28 ± 27.34 mg/dL, and the mean triglyceride (TG) level was 142.40 ± 66.95 mg/dl. In total, 67.5% of the study patients were on statin therapy. The mean left ventricular ejection fraction (LVEF) in this study was 57.23% ± 8.25% (range 36%–71%). The mean duration since CABG was 5.25 ± 4.04 years (range 2–21 years) (Table 1).

**Table 1:** Baseline and demographic data of the study group

	No. (40)	%
*Age: (years)*		
< 60	18	45.0
≥ 60	22	55.0
Mean ± SD	60.03 ± 9.09	
Range	42.0–82.0	
*Sex*		
Male	32	80.0
Female	8	20.0
*DM*		
Yes	27	67.5
No	13	32.5
*HTN*		
Yes	36	90.0
No	4	10.0
*BMI: (kg/m*^*2*^*)*		
Mean ± SD	28.89 ± 5.17	
Range	20.3–38.6	
*Smoking*		
Yes	12	30.0
No	28	70.0
*Dyslipidemia*		
Uncontrolled	27	67.5
Controlled	13	32.5
*LDL: (mg/dl)*		
Mean ± SD	84.28 ± 27.34	
Range	39.0–130.9	
*Triglycerides: (mg/dl)*		
Mean ± SD	142.40 ± 66.95	
Median (range)	122.5 (81.0–401)	
*Statin*		
Yes	27	67.5
No	13	32.5
*LVEF: (%)*		
Mean ± SD	57.23 ± 8.25	
Range	36.0–71.0	
*Duration since CABG: (years)*		
Mean ± SD	5.25 ± 4.04	
Median (range)	4.0 (2.0–21.0)	

DM, Diabetes mellitus; HTN, Hypertension; BMI, Body mass index; LVEF, Left ventricular ejection fraction

### CCTA and ICA findings in native vessels

In total, 124 native vessels were assessed using both CCTA and ICA and classified as 40 LADs, 40 left circumflex arteries (LCXs), 40 right coronary arteries (RCAs), and 4 ramus intermedius arteries. The posterior descending artery was assessed as part of the RCA or LCX according to origin. The main findings of CCTA for native LADs were as follows: one vessel showed an insignificant lesion, 15 vessels showed significant lesions, and 24 vessels were occluded. The same results were found on ICA indicating a good agreement between CCTA and ICA (kappa value = 1.0).

For native LCXs, CCTA revealed one normal vessel, one vessel with an insignificant lesion, 22 vessels with significant lesions, 12 totally occluded vessels, three vessels with diffuse atherosclerosis, and one non-evaluable vessel due to severe calcification, whereas ICA revealed two normal vessels, one vessel with an insignificant lesion, 23 vessel with significant lesions, 12 occluded vessels, and two vessels with diffuse atherosclerosis; therefore, agreement between CCTA and ICA was good (kappa value = 0.8).

CCTA findings for native RCAs revealed five normal vessels, one narrowed vessel with an insignificant lesion, 16 vessels with significant lesions, 15 totally occluded vessels, one vessel with diffuse atherosclerosis, and two non-evaluable vessels due to severe calcifications, whereas ICA revealed six normal vessels, one vessel with an insignificant lesion, 12 vessels with significant lesions,16 totally occluded vessels, three vessels with diffuse atherosclerosis, and two vessels that failed to be cannulated or visualized (abnormal posterior origin); therefore, the agreement between CCTA and ICA was accepted (kappa value = 0.67).

For the ramus intermedius, CCTA revealed two vessels with significant lesions and two totally occluded vessels, which were findings consistent with the results of ICA, indicating that the agreement between CCTA and ICA was good (kappa value = 1.0).

### CCTA and ICA findings in grafts

In our study, there were 97 evaluable grafts. The most frequently evaluated grafts were venous (59 SVGs) and arterial (38 LIMA grafts) grafts.

**Fig. 1:**
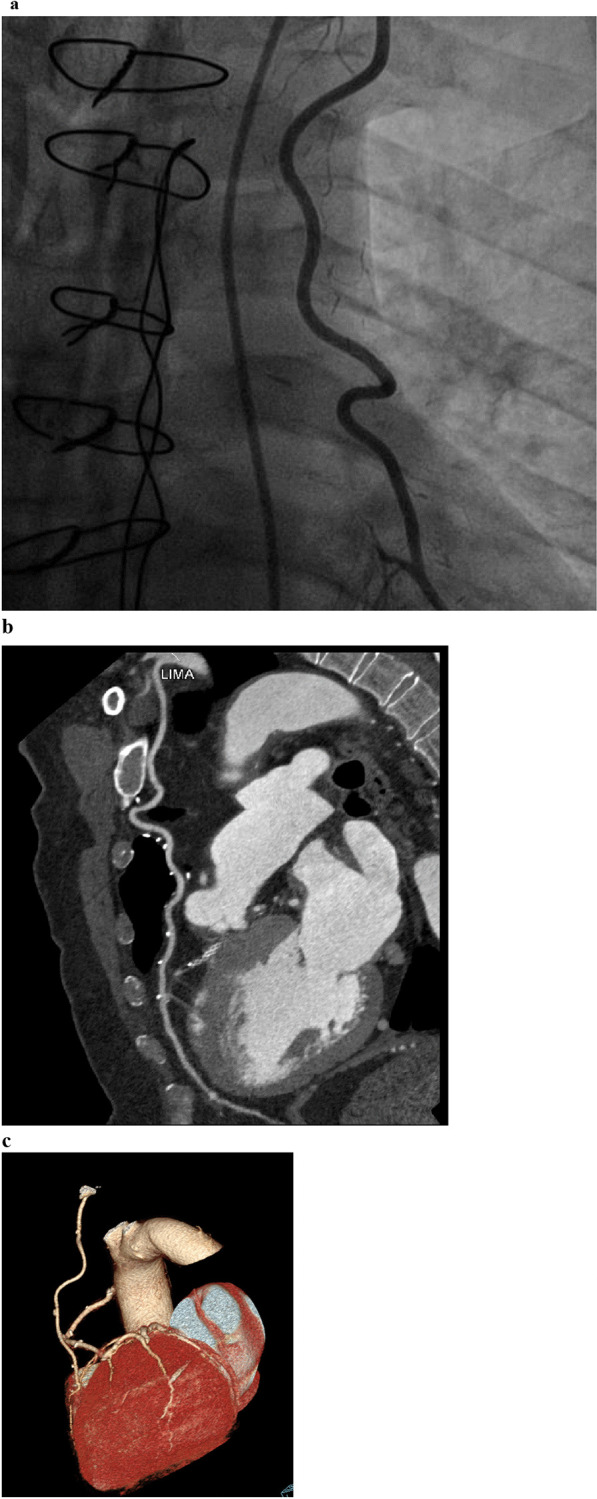
**a** ICA of a male patient post-CABG showing LIMA graft cannulation. **b** CCTA of the same post-CABG (curved MPR technique) showing a patent LIMA along its length with good anastomotic site and good distal opacification of LAD. **c** CCTA of the same patient post-CABG (volume-rendering images) showing patent LIMA to LAD, patent SVG to ramus intermedius, and patent SVG to RCA

We evaluated 38 LIMA grafts; CCTA detected 29 patent grafts, seven non-patent grafts, and two non-evaluable grafts due to artifacts caused by metallic clips, whereas ICA detected 28 patent grafts, four non-patent grafts, and six grafts that failed to be cannulated (Fig. 1).

In terms of venous grafts, 59 venous grafts were evaluated. CCTA revealed 20 patent grafts and 39 non-patent grafts, whereas ICA revealed only 12 patent grafts, 39 non-patent grafts, and eight grafts that failed to be cannulated.

In this study, we calculated the radiation dose in both CCTA and ICA as dose area product (DAP) (mGy cm^2^) and found a significantly lower radiation dose in CCTA than in ICA (1165.77 ± 123.54 vs. 47589.78 ± 6967.53, *p* < 0.001).

### Comparison between different risk factors-related graft patency

We compared the different factors affecting LIMA graft patency and our findings that variable cardiovascular risk factors such as age, sex, DM, hypertension, and smoking, have no significant influence on LIMA graft patency. Dyslipidemia, as evaluated by LDL and TG levels, had no significant association with LIMA graft patency. Moreover, no correlation was observed between LVEF and LIMA graft patency. Furthermore, no correlation was found between the mean duration since CABG and LIMA graft patency.

The severity of native vessel stenosis (LAD) has been evaluated, and all LIMA grafts were anastomosed to either significant stenosis or total occlusion, so we could not assess the relation between LAD stenosis severity and graft patency (Tables 2, 3).

**Table 2:** Comparison of the different risk factors affecting LIMA graft patency

	LIMA CT				*p*-value
	Patent		Non-patent		
	No	%	No	%	
*Age: (years)*					
< 60	11	37.9	5	71.4	0.204
≥ 60	18	62.1	2	28.6	
*Sex*					
Male	23	79.3	6	85.7	1.000
Female	6	20.7	1	14.3	
*DM*					
Yes	18	62.1	6	85.7	0.384
No	11	37.9	1	14.3	
*HTN*					
Yes	27	93.1	6	85.7	0.488
No	2	6.9	1	14.3	
*Smoking*					
Yes	10	34.5	2	28.6	1.000
No	19	65.5	5	71.4	
*BMI*					
Mean ± SD	27.84 ± 4.93		32.54 ± 5.74		0.035*
Range	20.9–38.6		20.3–38.2		
*Dyslipidemia*					
Uncontrolled	21	72.4	5	71.4	1.000
Controlled	8	27.6	2	28.6	
*Statin*					
Yes	19	65.5	4	57.1	0.686
No	10	34.5%	3	42.9%	
*LDL*					
Mean ± SD	86.40 ± 26.21		84.67 ± 29.86		0.880
Range	39.0–130.9		45.0–129.7		
*Triglyceride*					
Mean ± SD	140.14 ± 58.38		123.00 ± 35.05		0.459
Median (Range)	123.0 (81.0–308.0)		118.0 (90.0–189.0)		
*LVEF*					
Mean ± SD	57.10 ± 7.99		55.14 ± 9.49		0.577
Range	36.0–70.0		45.0–70.0		
*Duration since CABG: (years)*					
Mean ± SD	4.59 ± 2.88		6.29 ± 4.23		0.361
Range	4.0 (2.0–13.0)		6.0 (2.0–13.0)		

DM, Diabetes mellitus; HTN, Hypertension; BMI, Body mass index; LVEF, Left ventricular ejection fraction

**Table 3:** Native vessel lesion in correlation with LIMA graft patency

LAD	LIMA CT				*p*-value
	Patent		Non-patent		
	No	%	No	%	
Non-significant lesion	0	0.0	0	0.0	1.000
Significant lesion	11	37.9	2	28.6	
Total occlusion	18	62.1	5	71.4	

Regarding SVG patency, we examined the same determinants of graft patency and found no association between graft patency and different risk factors, including age, sex, DM, hypertension, obesity, and smoking, and SVG patency and the severity of native vessel lesion, but we found a significant association between SVG patency and LDL and TG levels (LDL level: 68.02 ± 29.73 mg/dL vs. 104.45 ± 15.24 mg/dL; *p* = 0.002) (TG level: 109.78 ± 22.97 mg/dL vs. 127.00 ± 22.03 mg/dL; *p* = 0.05). Furthermore, lower LVEF values have been associated with lower SVG patency (62.83% ± 6.11% (range, 55.0%–70.0%) vs. 55.4% ± 7.45% (range 40.0%–67.0%; *p* = 0.04)) (Tables 4, 5, 6, 7).

**Table 4:** Comparison of the different risk factors affecting Saphenous vein graft patency

	SVG-LCX-OM CT				*p*-value
	Patent		Non-patent		
	No	%	No	%	
*Age: (years)*					
< 60	4	66.7	8	44.4	0.640
≥ 60	2	33.3	10	55.6	
*Sex*					
Male	6	100.0	15	83.3	0.546
Female	0	0.0	3	16.7	
*DM*					
Yes	6	100.0	11	61.1	0.130
No	0	0.0	7	38.9	
*HTN*					
Yes	6	100.0	17	94.4	1.000
No	0	0.0	1	5.6	
*Smoking*					
Yes	2	33.3	5	27.8	1.000
No	4	66.7	13	72.2	
*BMI*					
Mean ± SD	25.35 ± 3.95		29.11 ± 5.72		0.152
Range	22.4–32.0		20.3–38.6		
*Dyslipidemia*					
Uncontrolled	3	50.0	15	83.3	0.139
Controlled	3	50.0	3	16.7	
*Statin*					
Yes	6	100.0	12	66.7	0.277
No	0	0.0	6	33.3	
*LDL*					
Mean ± SD	72.47 ± 23.06		96.29 ± 25.44		0.055
Range	52.4–110.8		45.0–130.9		
*Triglyceride*					
Mean ± SD	177.17 ± 112.18		126.39 ± 49.12		0.142
Median (Range)	147.0 (102.0–401.0)		115.5 (81.0–308.0)		
*LVEF*					
Mean ± SD	62.83 ± 6.11		55.44 ± 7.45		0.040*
Range	55.0–70.0		40.0–67.0		
*Duration since CABG: (years)*					
Mean ± SD	3.17 ± 1.33	5.67 ± 5.04			0.231
Range	3.0 (2.0–5.0)	4.0 (2.0–21.0)			

DM, Diabetes mellitus; HTN, Hypertension; BMI, Body mass index; LVEF, Left ventricular ejection fraction

**Table 5:** Native vessel lesion in correlation with Saphenous vein graft patency

LCX	SVG-LCX-OM CT				*p*-value
	Patent		Non-patent		
	No	%	No	%	
Non-significant lesion	0	0.0	0	0.0	0.339
Significant lesion	4	80.0	9	50.0	
Total occlusion	1	20.0	9	50.0

**Table 6:** Comparison of the different risk factors affecting Saphenous vein graft patency

	SVG-RCA CT				*p*-value
	Patent		Non-patent		
	No	%	No	%	
*Age: (years)*					
< 60	4	44.4	5	41.7	1.000
≥ 60	5	55.6	7	58.3	
*Sex*					
Male	8	88.9	10	83.3	1.000
Female	1	11.1	2	16.7	
*DM*					
Yes	6	66.7	8	66.7	1.000
No	3	33.3	4	33.3	
*HTN*					
Yes	8	88.9	11	91.7	1.000
No	1	11.1	1	8.3	
*Smoking*					
Yes	2	22.2	6	50.0	0.367
No	7	77.8	6	50.0	
*BMI*					
Mean ± SD	30.42 ± 5.29		27.68 ± 5.60		0.269
Range	22.9–38.2		20.3–35.6		
*Dyslipidemia*					
Uncontrolled	3	33.3	12	100.0	0.002*
Controlled	6	66.7	0	0.0	
*Statin*					
Yes	6	66.7	7	58.3	1.000
No	3	33.3	5	41.7	
*LDL*					
Mean ± SD	68.02 ± 29.73		104.45 ± 15.24		0.002*
Range	45.0–129.0		78.6–130.9		
*Triglyceride*					
Mean ± SD	109.78 ± 22.97		127.00 ± 22.03		0.050*
Median (Range)	96.0 (90.0–154.0)		127.5 (102.0–159.0)		
*LVEF*					
Mean ± SD	57.33 ± 7.25		58.75 ± 8.08		0.683
Range	45.0–67.0		50.0–70.0		
*Duration after CABG: (years)*					
Mean ± SD	5.78 ± 4.74		6.75 ± 5.38		0.540
Range	3.0 (2.0–13.0)		5.0 (2.0–21.0)		

DM, Diabetes mellitus; HTN, Hypertension; BMI, Body mass index; LVEF, Left ventricular ejection fraction

**Table 7:** Native vessel lesion in correlation with Saphenous vein graft patency

RCA	SVG-RCA CT				*p*-value
	Patent		Non-patent		
	No	%	No	%	
Non-significant lesion	0	0.0	0	0.0	0.642
Significant lesion	2	22.2	5	41.7	
Total occlusion	7	77.8	7	58.3	

### CCTA versus ICA in graft assessment

In this study, six LIMA grafts failed to be cannulated by ICA and were evaluated using CCTA, two grafts were patent, three grafts were non-patent, and one graft was non-evaluable due to metallic clips. Furthermore, eight venous grafts failed to be cannulated by ICA and were evaluated using CCTA. We found two patent grafts, two grafts with significant stenosis, and four totally occluded grafts.

## Discussion

Graft patency is the assumed mechanism for long-term benefits of coronary artery bypass grafting (CABG) [10]. Graft failure is considered a complex and multifactorial event occurring in significant percentage of CABG conduits [11]. It is thought to be a strong indicator for future cardiac events as myocardial infarction, repeat revascularization, and death [12].

However, recurrence of ischemic symptoms may happen due to graft failure, the progression of native coronary artery atherosclerosis, or a combination of both [13].

With the increased survival of CABG patients, there is a greater interest in the best diagnostic procedures for patients who present with ischemic symptoms following surgery [14].

ICA remains the gold standard diagnostic test for evaluating symptomatic post-CABG patients However, this invasive procedure carries the risk of cardiac arrhythmia, stroke and vascular complications like dissection, bleeding, pseudo-aneurysm [15]. CCTA has emerged as an alternative to ICA with comparable sensitivities (81%) and specificities (91%) [16].

This study used CCTA and ICA to assess graft patency and native vessel lesions in 40 symptomatic patients following CABG and correlated the different risk factors with graft patency.

Regarding the patency of venous grafts assessed using CCTA, most venous grafts were non-patent, representing 66.1% of these grafts with a mean duration after CABG of 5.25 ± 4.04 years. This was consistent with the findings of a large Veterans Affairs trial by Goldman et al. who reported a 10-year patency rate for SVGs of 61%. Meanwhile, most arterial grafts (LIMA) assessed using CCTA were patent (76.3%), which is less than the patency rate stated by Goldman et al. for LIMA grafts 10 years after CABG (85%). This can be explained by the wide range of duration after CABG, ranging from 2 to 21 years, and the frequency of smoking and uncontrolled dyslipidemia in our cohort [17].

We studied the variable cardiovascular risk factors, such as age, sex, hypertension, DM, and smoking status, and we found that they are not predictive for long-term graft occlusion; this finding is consistent with Harskamp et al. who evaluated the determinants of LIMA graft stenosis or occlusion in a study of 1539 CABG patients undergoing angiographic follow-up from the PREVENT IV trial [18].

Regarding DM, 67.5% of the patients in this study had DM. According to Harskamp et al., the absence of DM was the only patient-specific predictor of LIMA graft failure as patients with DM might have more severe proximal coronary artery stenosis [18]. Moreover Shah et al. found in their study which included 3715 angiograms that DM did not affect graft patency [19].

In contrast, Deb et al. reported that compared with non-diabetic patients, SVG occlusion was higher in diabetic patients 5 years or more after CABG [20]. However, this study included patients who were followed up 1 year after CABG, so the percentage of patent grafts was larger.

LDL and TG levels were evaluated, and we found a significant association between LDL, TG levels, and SVG patency. This is consistent with the results of Hata et al. who noted yellow plaque and thrombus using intracoronary angioscopy in the vein grafts of patients with high LDL levels (more than 100 mg/dL) one year after surgery. However, they found that plaque and thrombus were absent in patients with low LDL levels (less than 80 mg/dL), suggesting that aggressive lipid-lowering treatment following CABG may prevent the development of SVG failure [21].

The diagnostic performance of CCTA for assessing both arterial and venous grafts as well as native coronary arteries was compared with that of ICA. We used CCTA to evaluate the non-cannulated grafts and found that one-third of them were patent. Krysztofiak et al*.* have reported similar findings, stating that only 79% of the LIMA grafts under study were cannulated [22]. The use of CCTA to confirm patency of LIMA graft may reduce the need for selective cannulation during ICA, which sometimes carries the risk of dissection and difficult cannulation because of its acute origin from the subclavian artery, smaller lumen, and large mediastinal course. LIMA dissection may cause severe myocardial damage that may require emergency cardiac surgery (redo CABG) or complex percutaneous coronary intervention [23]. Secondly, access is gained through either femoral or left radial route. The use of left radial access adds to the difficulty of the procedure resulting in prolonged procedure time and more contrast agent use. [24]. The trans-femoral access is also hampered by the vascular site complications caused by arterial puncture. Moreover, due to the proximity of the LIMA origin to the left vertebral artery, there is risk of ischemic stroke [22].

In this study, eight non-cannulated venous grafts were found patent using CCTA, and this is of course a game changer in the planned revascularization strategy.

Our results are concordant with the results of Weustink et al. who stated that MSCT has sensitivity of 100% for the detection of significant per segment graft obstruction and 95% for the detection of significant distal runoffs lesions [25].

In this study, the two modalities had good agreement in detecting significant stenosis and total occlusion of native vessels with an average limit of agreement of 0.8 (kappa value). Similar results were reported by Weustink et al. and Radwan et al. using 256-slice dual-source MDCT who stated per-patient, per-vessel, and per-segment CCTA accuracy of 95%, 88%, and 96%, respectively [25, 26].

The radiation dose was calculated for both CCTA and ICA, and we found that the radiation dose in CCTA was significantly lower, which is consistent with the findings of Koplay et al. using 128-slice MDCT [27].

In this study, we used Siemens Somatom Definition 128-slice CT scanner with broad detector array and dual-source high helical pitch scanning to capture whole entire heart in a single beat, which resulted in a significant dose reduction. Its rotation speeds are faster, which improved the temporal resolution. Using ECG-based tube current modulation with a retrospective ECG-gated technique to reduce the applied tube current by 20%, iterative reconstruction techniques achieved 30–41% reduction in radiation dose.

## Conclusions

In conclusion, CCTA has a comparable diagnostic accuracy in assessing bypass grafts and native coronary arteries to ICA and thus provides a non-invasive reliable tool for evaluating patients following CABG with less radiation dose. As a result, we advocate for the use of CCTA at the outset to divert management away from ICA, with expected significant reduction of patients’ exposure to contrast material and radiation.

## Limitations

1. Because this study only included symptomatic patients 1 year after CABG, we detected a higher percentage of graft failure.
2. Due to the coronavirus disease 2019 pandemic, the sample size was limited with restriction to some elective investigations while maintaining the emergency procedure.

## Data Availability

We confirm that all patients’ data are available including the diagnostic studies and excel sheets.

## Abbreviations

BMI: Body mass index
CABG: Coronary artery bypass grafting
CAD: Coronary artery disease
CCTA: Coronary computed tomography angiography
DLP: Dose length product
DM: Diabetes mellitus
ICA: Invasive coronary angiography
IMA: Internal mammary artery
LAD: Left anterior descending artery
LCX: Left circumflex artery
LIMA: Left internal mammary artery
LVEF: Left ventricular ejection fraction
MDCT: Multidetector computed tomography
MIP: Maximum intensity projection
MPR: Multiplanar reformatted
NPV: Negative predictive value
PCI: Percutaneous coronary intervention
PPV: Positive predictive value
SVG: Saphenous vein graft

## References

[1] Masroor M, Ahmad A, Wang Y, Dong N (2023). Assessment of the graft quality and patency during and after coronary artery bypass grafting. Diagnostics.

[2] Head SJ, Milojevic M, Taggart DP, Puskas JD (2017). Current practice of state-of-the-art surgical coronary revascularization. Circulation.

[3] Di Lazzaro D, Crusco F (2017). CT angio for the evaluation of graft patency. J Thorac Dis.

[4] Hsiao EM, Rybicki FJ, Steigner M (2010). CT coronary angiography: 256-slice and 320-detector row scanners. Curr Cardiol Rep.

[5] Al-Hijji MA, Lennon RJ, Gulati R, El Sabbagh A, Park JY, Crusan D, Kanwar A, Behfar A, Lerman A, Holmes DR (2019). Safety and risk of major complications with diagnostic cardiac catheterization. Circ Cardiovasc Interv.

[6] Beirne AM, Rathod KS, Castle E, Andiapen M, Richards A, Bellin A, Hammond V, Godec T, Moon JC, Davies C (2021). The BYPASS-CTCA study: the value of computed tomography cardiac angiography (CTCA) in improving patient-related outcomes in patients with previous bypass operation undergoing invasive coronary angiography: study protocol of a randomised controlled trial. Ann Transl Med.

[7] Koplay M, Guneyli S, Akbayrak H, Demir K, Sivri M, Avci A, Erdogan H, Paksoy Y (2016). Diagnostic accuracy and effective radiation dose of high pitch dual source multidetector computed tomography in evaluation of coronary artery bypass graft patency. Wien Klin Wochenschr.

[8] Andreini D, Pontone G, Mushtaq S, Annoni A, Formenti A, Bertella E, Parolari A, Agostoni P, Bartorelli A, Ballerini G (2012). Diagnostic performance of two types of low radiation exposure protocol for prospective ECG-triggering multidetector computed tomography angiography in assessment of coronary artery bypass graft. Int J Cardiol.

[9] Sahiner L, Canpolat U, Yorgun H, Hazrolan T, Karçaaltncaba M, Sunman H, Kaya EB, Aytemir K, Oto A (2012). Diagnostic accuracy of dual-source 64-slice multidetector computed tomography in evaluation of coronary artery bypass grafts. J Investig Med.

[10] Gaudino M, Sandner S, An KR, Dimagli A, Di Franco A, Audisio K, Harik L, Perezgrovas-Olaria R, Soletti G, Fremes SE (2023). Graft failure after coronary artery bypass grafting and its association with patient characteristics and clinical events: a pooled individual patient data analysis of clinical trials with imaging follow-up. Circulation.

[11] Gaudino M, Antoniades C, Benedetto U, Deb S, Di Franco A, Di Giammarco G, Fremes S, Glineur D, Grau J, He GW (2017). Mechanisms, consequences, and prevention of coronary graft failure. Circulation.

[12] Hess CN, Lopes RD, Gibson CM, Hager R, Wojdyla DM, Englum BR, Mack MJ, Califf RM, Kouchoukos NT, Peterson ED (2014). Saphenous vein graft failure after coronary artery bypass surgery: insights from PREVENT IV. Circulation.

[13] Beerkens FJ, Claessen BE, Mahan M, Gaudino MFL, Tam DY, Henriques JPS, Mehran R, Dangas GD (2022). Contemporary coronary artery bypass graft surgery and subsequent percutaneous revascularization. Nat Rev Cardiol.

[14] Chan M, Ridley L, Dunn DJ, Tian DH, Liou K, Ozdirik J, Cheruvu C, Cao C (2016). A systematic review and meta-analysis of multidetector computed tomography in the assessment of coronary artery bypass grafts. Int J Cardiol.

[15] Gabriel J, Klimach S, Lang P, Hildick-Smith D (2015). Should computed tomography angiography supersede invasive coronary angiography for the evaluation of graft patency following coronary artery bypass graft surgery?. Interact Cardiovasc Thorac Surg.

[16] Saade C, Fakhredin RB, El Achkar B, Ghieh D, Mayat A, Abchee A, Refaat M, Ismail H, El-Rayess H, Karout L (2019). Coronary artery anomalies and associated radiologic findings. J Comput Assist Tomogr.

[17] Goldman S, Zadina K, Moritz T, Ovitt T, Sethi G, Copeland JG, Thottapurathu L, Krasnicka B, Ellis N, Anderson RJ (2004). Long-term patency of saphenous vein and left internal mammary artery grafts after coronary artery bypass surgery: results from a Department of Veterans Affairs Cooperative Study. J Am Coll Cardiol.

[18] Harskamp RE, Alexander JH, Ferguson TB, Hager R, Mack MJ, Englum B, Wojdyla D, Schulte PJ, Kouchoukos NT, de Winter RJ (2016). Frequency and predictors of internal mammary artery graft failure and subsequent clinical outcomes: insights from the project of ex-vivo vein graft engineering via transfection (PREVENT) IV trial. Circulation.

[19] Shah PJ, Gordon I, Fuller J, Seevanayagam S, Rosalion A, Tatoulis J, Raman JS, Buxton BF (2003). Factors affecting saphenous vein graft patency: clinical and angiographic study in 1402 symptomatic patients operated on between 1977 and 1999. J Thorac Cardiovasc Surg.

[20] Deb S, Singh SK, Moussa F, Tsubota H, Une D, Kiss A, Tomlinson G, Afshar M, Sless R, Cohen EA et al (2014) The long-term impact of diabetes on graft patency after coronary artery bypass grafting surgery: a substudy of the multicenter Radial Artery Patency Study. J Thorac Cardiovasc Surg 148(4):1246–1253; discussion 1253 10.1016/j.jtcvs.2014.06.057 25109754

[21] Hata M, Takayama T, Sezai A, Yoshitake I, Hirayama A, Minami K (2009). Efficacy of aggressive lipid controlling therapy for preventing saphenous vein graft disease. Ann Thorac Surg.

[22] Krysztofiak T, Ahmad F, Adams J, Stobo DB, Good R, Byrne J (2020). The value of non-invasive computed tomography coronary angiography in imaging patients with coronary artery bypass grafts. Scott Med J.

[23] Khan Z, Latif F, Dasari TW (2014). Internal mammary artery graft dissection: a case-based retrospective study and brief review. Tex Heart Inst J.

[24] Michael TT, Alomar M, Papayannis A, Mogabgab O, Patel VG, Rangan BV, Luna M, Hastings JL, Grodin J, Abdullah S (2013). A randomized comparison of the transradial and transfemoral approaches for coronary artery bypass graft angiography and intervention: the RADIAL-CABG trial (RADIAL versus femoral access for coronary artery bypass graft angiography and intervention). JACC Cardiovasc Interv.

[25] Weustink AC, Nieman K, Pugliese F, Mollet NR, Meijboom WB, van Mieghem C, ten Kate GJ, Cademartiri F, Krestin GP, de Feyter PJ (2009). Diagnostic accuracy of computed tomography angiography in patients after bypass grafting: comparison with invasive coronary angiography. JACC Cardiovasc Imaging.

[26] Radwan H, Kandil N, Elshaer M, Abd-Elkader A (2019). Diagnostic accuracy of 256 slices computed tomography coronary angiography in post coronary artery bypass graft Egyptian patients. J Indian Coll Cardiol.

[27] Koplay M, Erdogan H, Avci A, Sivri M, Demir K, Guler I, Demir LS, Paksoy Y (2016). Radiation dose and diagnostic accuracy of high-pitch dual-source coronary angiography in the evaluation of coronary artery stenoses. Diagn Interv Imaging.

